# Automated dual olfactory device for studying head/tail chemosensation in *Caenorhabditis elegans*

**DOI:** 10.1063/5.0187441

**Published:** 2024-04-18

**Authors:** Shadi Karimi, Asaf Gat, Costanza Agazzi, Meital Oren-Suissa, Michael Krieg

**Affiliations:** 1 ICFO—Institut de Ciències Fotòniques, Castelldefels, The Barcelona Institute of Science and Technology, Barcelona, Spain; 2 Mechanical Engineering Department, Technical University of Catalonia, Terrassa, Barcelona, Spain; 3 Department of Brain Sciences, Weizmann Institute of Science, Rehovot, Israel

## Abstract

The correct interpretation of threat and reward is important for animal survival. Often, the decisions underlying these behavioral programs are mediated by volatile compounds in the animal's environment, which they detect and discriminate with specialized olfactory neurons along their body. *Caenorhabditis (C.) elegans* senses chemical stimuli with neurons located in the head and the tail of the animal, which mediate either attractive or aversive behaviors. How conflicting stimuli are processed in animals navigating different chemical gradients is poorly understood. Here, we conceived, created, and capitalized on a novel microfluidic device to enable automated and precise stimulation of head and tail neurons, either simultaneously or sequentially, while reading out neuronal activity in sensory and interneurons using genetically encoded calcium indicators. We achieve robust and programmable chemical pulses through the modulation of inlet pressures. To evaluate the device performance, we synchronized the flow control with microscopy data acquisition and characterized the flow properties in the fabricated devices. Together, our design has the potential to provide insight into the neural circuits and behavior of *C. elegans* simulating the experience of natural environments.

## I. INTRODUCTION

How organisms navigate and interact with their environment depends on the full integration of all sensory information. Such cues are mechanical, optical, thermal, and chemical in nature. Olfaction, the sense of smell, is a remarkable sensory modality that plays a pivotal role in our daily lives and profoundly influences our behaviors and decisions. Beyond its fundamental role in detecting and identifying odors in our environment, olfaction extends its influence on complex processes, such as threat detection and reward seeking. The detection of noxious odors, such as the smell of smoke signaling a fire or the scent of predators lurking nearby, can trigger rapid and life-saving behaviors. Conversely, olfaction is equally vital for reward processing. From the pleasurable aroma of a favorite meal to the alluring scent of a potential mate, odors have the power to elicit profound emotional and physiological responses. Unraveling the neural mechanisms by which olfactory cues inform reward-seeking behaviors can provide valuable insights into addiction, decision-making, and even therapeutic interventions for conditions like obesity and substance abuse.

Often, organisms experience a potpourri of different odors that impinges on different chemosensors and need to process conflicting information to make informed decisions about threat and reward. *Caenorhabditis elegans* is a free living terrestial nematode, which has chemosensory neurons in the head and in the tail, requiring information from both of them to initiate a behavioral response (Hilliard *et al.*, 2002). However, how the same odorant that acts simultaneously on these separate sensory neurons does not generate a contradictory behavioral response (Gat *et al.*, 2023) and how organisms process chemicals and olfactory cues that carry conflicting information is not particularly well understood. We have recently shown that the single rich club interneuron AVA receives both excitatory glutamatergic signals from head sensory neuron ASH and inhibitory signals from tail sensory neurons PHA and PHB in response to high osmolarity. We found that the differential activation of AVA arises from the unique distribution of glutamate-gated receptors, with excitatory and inhibitory receptors positioned along different segments of AVA's structure (Gat *et al.*, 2023). AVA, thus, serves as an integrator, processing these spatially distinct and contrasting cues to generate an output that guides the animal's behavioral decisions. Whether or not these findings represent a universal cellular mechanism that underlies spatial computation within the nervous system is an outstanding question.

*C. elegans*, with its optically transparent body, well-defined nervous system, and powerful genetics is an excellent system for examining chemosensation. It is now established that conflicting sensory stimuli affect the primary behavior of the animals and skew their threat/reward decision-making. For example, starved animals are more likely to inhibit their response to a noxious stimulus that otherwise causes an aversive response in well-fed animals. The neuronal correlate for this prioritization, however, is currently under intense study, complicated by the fact that *C. elegans* has chemosensors that are located in the head and in the tail (Hilliard *et al.*, 2002; Pechuk *et al.*, 2022; and Gat *et al.*, 2023). For instance, the tail sensors might play a role in suppressing or modulating the responses of the nose sensors, leading to intricate and interrelated mechanisms of sensory regulation (Bargmann, 2012). Exploring these complex interactions and uncovering the underlying regulatory mechanisms are key intellectual challenges in studying olfactory stimuli and chemosensation in organisms.

However, performing microscopic recordings of neuronal calcium transients in *C. elegans* under controlled application of external stimuli is challenging, primarily due to the intrinsic locomotory activity of the animal. Early experiments involved simple perfusion of glued animals with solutions delivered through micro-manipulator operated capillary, fluid-filled pipettes (Hilliard *et al.*, 2005). Nevertheless, both gluing and manual immobilization procedures are time-consuming, labor-intensive, lack the temporal precision for rapid stimulation, and are invasive as the animal cannot be recovered after the interrogation. Additionally, the toxicity of the organic glue to the worm or how it influences neuronal activity is difficult to determine (Chronis *et al.*, 2007). The adoption of microfluidic technologies offers a great replacement for conventional experiments, providing automated manipulation, high throughput assays, and precise handling of both animals and liquids. This automation allows for experimental standardization by minimizing the need for manual labor. Conveniently, due to their size, *C. elegans* is well-suited for microfluidic applications. The first devices accomplished tasks such as noninvasive animal immobilization without the use of tissue adhesives (Hulme *et al.*, 2007) and long-term culture for longitudinal studies during development (Keil *et al.*, 2017) and ageing (Xian *et al.*, 2013). More complex designs integrate animal immobilization with the presentation of defined stimuli and simultaneous microscopy to investigate the neuronal response to chemicals (Chronis *et al.*, 2007), electrical fields (Rezai *et al.*, 2010), gasses (Gray *et al.*, 2005; Hu *et al.*, 2015), thermal (Gonzales *et al.*, 2019), and mechanical stimuli (Cho *et al.*, 2017; Nekimken *et al.*, 2017; Setty *et al.*, 2022; Porta-de-la Riva *et al.*, 2023; and Sanfeliu-cerdán *et al.*, 2023). Several devices have been developed and deployed to investigate how *C. elegans* senses olfactory stimuli. Among those, various microfluidic platforms afforded behavioral studies (Albrecht and Bargmann, 2011) or serial/parallel immobilization of animals for high-resolution imaging of neuronal activity under the influence of an olfactory stimulus (Bazopoulou *et al.*, 2017; Chronis *et al.*, 2007; Chung *et al.*, 2020; Lin *et al.*, 2023; Reilly *et al.*, 2017; Rouse *et al.*, 2018; and Zimmer *et al.*, 2009). Through the incorporation of automated image acquisition and data analysis strategies, along with the use of genetically expressed fluorescent probes, these platforms have extensively leveraged *C. elegans* advantages for *in vivo* genetic and compound screens and permit the construction of well-controllable environments (Chronis *et al.*, 2007). Specifically, the platform pioneered by Chronis *et al.* has emerged as the benchmark tool for evaluating chemosensory neuronal activity in *C. elegans*. Its user-friendly interface and capability to subject the organism to swift stimulus sequences of up to 5 Hz have solidified its status as the standard in the field (Chronis *et al.*, 2007). To extend the original design for the controlled delivery of multiple stimuli, Rouse *et al.* developed a platform to deliver programmable sequences of up to four chemicals with sub-second resolution, while simultaneously monitoring *C. elegans* neuronal activity (Rouse *et al.*, 2018). However, most of these microfluidic delivery systems for olfactory stimuli have been used to stimulate a single worm either to the head or, if the animal was inserted in reverse, to the tail (Salzberg *et al.*, 2020). No device permitted the controlled application of an olfactory stimulus, sequentially or simultaneously, to both the head and tail of the same animal in an automated manner.

Here, we develop a new microfluidic device to simultaneously stimulate the head and tail while monitoring neural activity with high-resolution microscopy under the influence of different olfactory stimuli. This novel design offers several advantages: (1) automatic exposure of the head and tail simultaneously, (2) faster experimental setup even when exposing solely the head or the tail, by quickly loading worms regardless of their head-tail orientation, (3) precise imaging of neural activity, (4) a programmable platform synced with the stimulating system for imaging the neuronal responses in real-time, (5) dedicated devices with trapping channels adapted for both hermaphrodites and males, (6) optimized loading chamber for fast sequential loading and removal of animals that can be conveniently parked in the channel ante-chamber, ideal for recording stimulus-evoked responses with high throughput, and (7) alleviation of the need to anesthetize animals during the stimulus protocol.

## II. RESULTS

### A. The device design

**FIG. 1.**
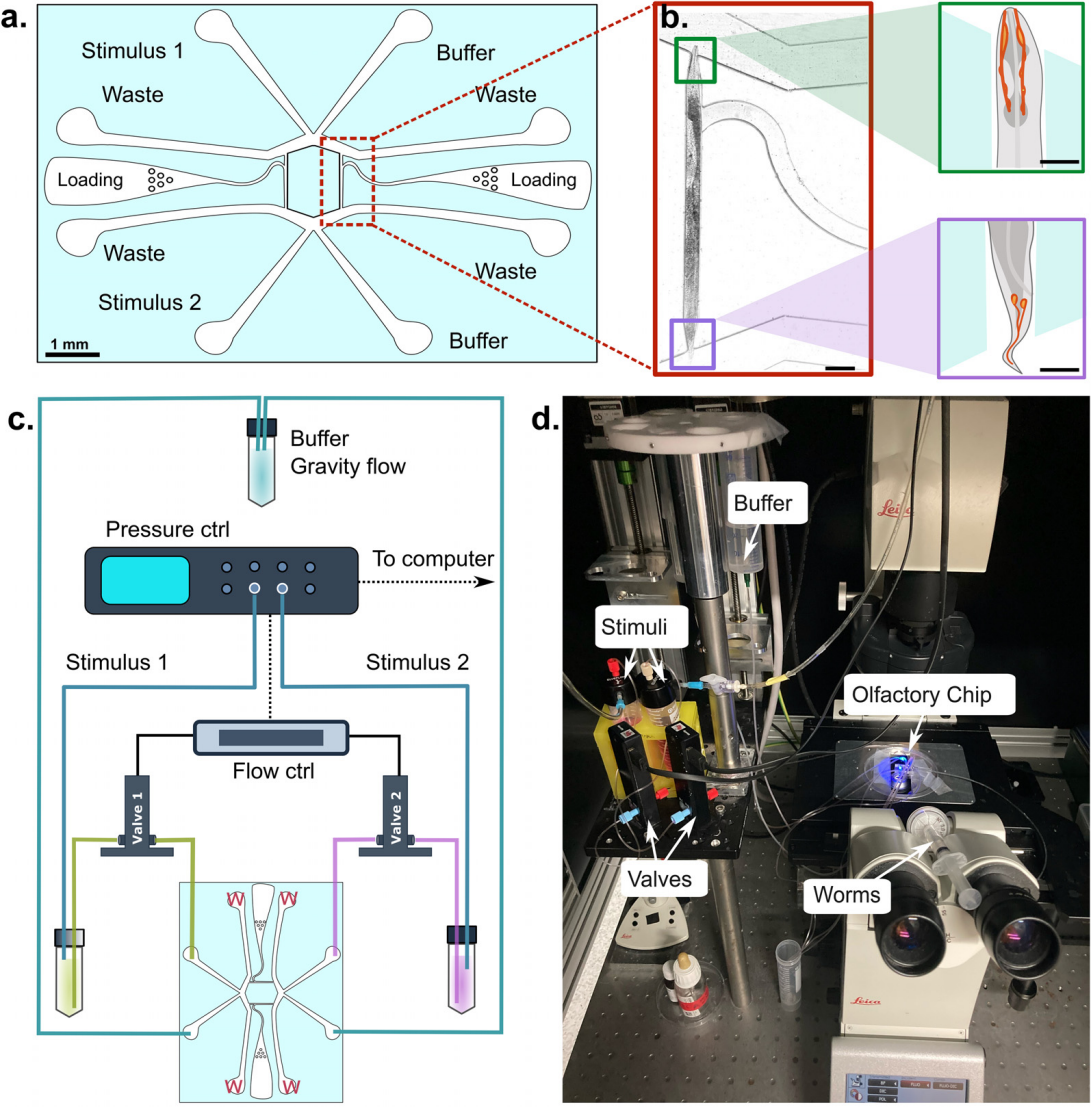
Design of the device and experimental setup. (a) Layout of the device with the channels for buffer perfusion, stimulus delivery, animal loading, and waste collection. (b). Brightfield micrograph of a *C. elegans* immobilized in the trapping channel with its nose and tail protruding into the flow channels. Scale bar = 100 μm. Squares to the right show a schematic of the olfactory neurons in the head and tail of the animal. Scale bar for both insets = 50 μm. (c). Layout of the different connections from the stimulus and buffer reservoir to the controller and the channels. The buffer reservoir (e.g., M9) is mounted on a motorized linear stage to adjust the gravity flow and connects directly to the chip, enabling a constant flow around the animal. The stimulus reservoirs are pressurized with a pressure controller and connected to the chip through a normally closed solenoid valve. Opening of the valve displaces the buffer and exposes either the nose or the tail to the stimulus solution. The waste channels are indicated with the letter “W.” (d) Photograph of the setup with the indicated components.

Our goal was to develop an automated, easy-to-operate microfluidic platform for stimulating both the head and tail of *C. elegans* while monitoring their neuronal activity in response to stimulating odorants. The platform consisted of the microfluidic device mounted on top of a confocal or epifluorescence microscope to visualize changes in neurophysiology upon stimulation, with the help of a neuronal activity indicator. During this study, we explored different designs to deliver a chemical stimulus to either the head or tail of a microfluidically trapped animal (supplementary material Fig. 1). Finally, we prioritized a design that afforded a separate delivery of two different stimuli to either head, tail, or both [Fig. 1(a)]. To allow for fast perfusion of the stimulant, we adapted the four-flow system of the “olfactory chip” (Chronis *et al.*, 2007), to switch between a laminar buffer flow and the stimulant solution. The final design was symmetric and we found it helpful to include two trapping channels with separate inlets, which facilitates sequential imaging of different animals with the same chip assembly in the unfavorable case that the trapping channel gets clogged during animal loading/removal procedure. In that way, a single device can be reused and does not need to be discarded.

Loading the animals into the microfluidic device proved to be the most challenging aspect of the experimental design, as the head and tail needed to be presented into two separate flow channels. To avoid damage to the body of the animal while ensuring proper placement within the trapping channel, we implemented a curved entrance to facilitate gentle and effective placement within the trapping channel.

The final design was optimized with a 1 mm long trapping channel, tailored to fit age-synchronized day one adult hermaphrodites (length *L* ≈1.16±0.16 mm, N = 3 replicates, n = 15 animals each) with their head and tail exposed to the flow channel. This length is representative of wild-type and many transgenic animals; thus, little variation can be expected between different experiments and mutants that do not affect body morphology (Malaiwong *et al.*, 2023). To accommodate the shape and size of the worm's head and tail, the trapping channel had tapered ends. With this design, the trap consistently presented the nose and tail with their respective sensory neuron endings to the flow channel [Fig. 1(b)], while flow control was achieved with an automated pressure controller and pair of solenoid valves connected to the appropriate and buffer channels [Methods and Figs. 1(c) and 1(d)]. We should also point out that trapping was significantly easier than with other designs, as all the orientations of the animal in the trapping channel did not matter for the experiment. In other words, regardless of orientation, the protocol could be executed successfully. Furthermore, similar to other microfluidic designs, the animals could be recovered from the trapping channel and “rescued” from the chip for longitudinal experiments. Importantly, to cater to *C. elegans* males, which are slightly thinner and shorter, we included a device iteration with trapping channels that are 30 μm wide and 700 μm long. These male-specific channels will also fit smaller hermaphrodites, e.g., mutants or L4 larval stage. However, mutant animals with suspected defects in odor sensation that also show defects in body size require a redesign of the trapping channel for proper investigation.

### B. Calibration of stimulus perfusion

**FIG. 2.**
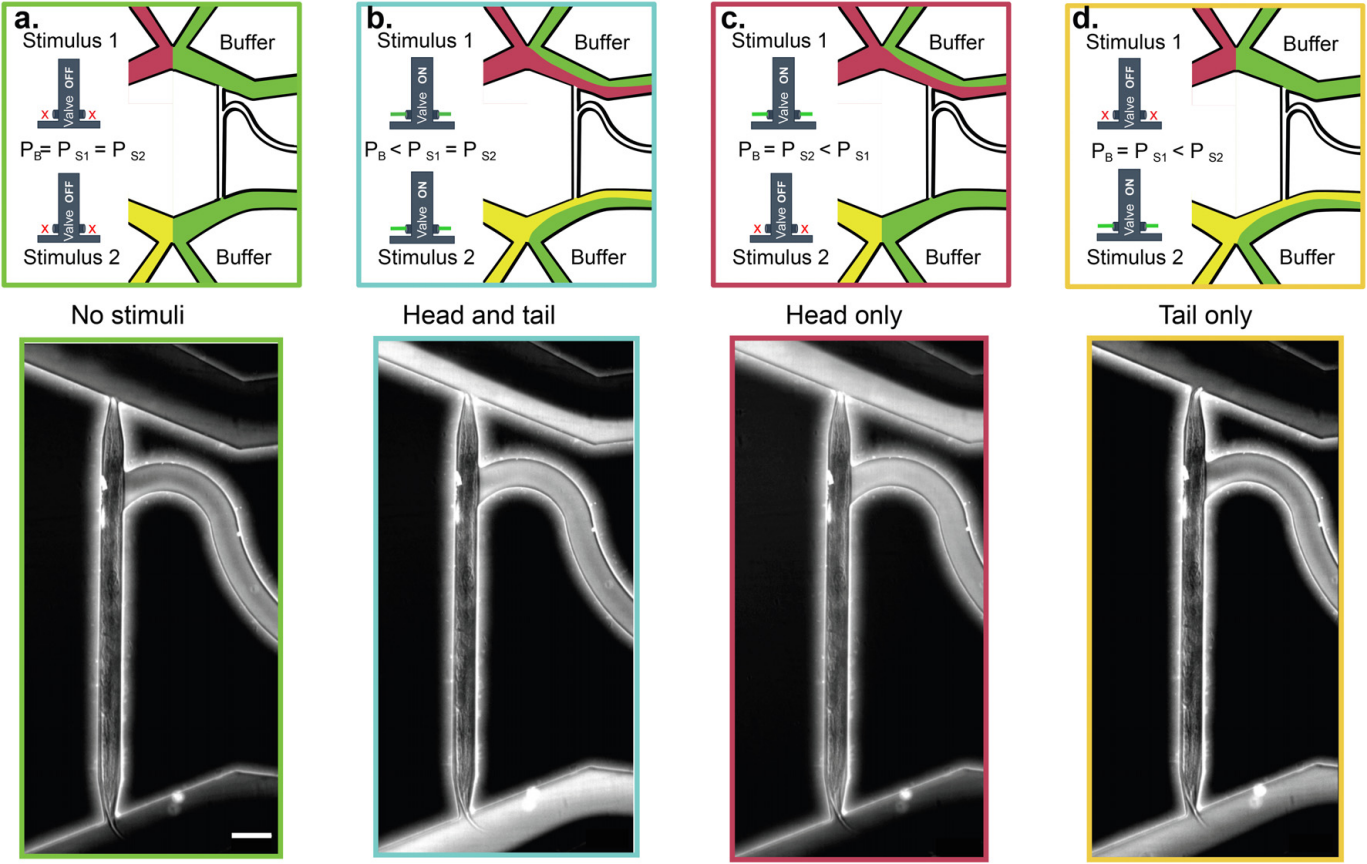
Operation of the dual olfactory device. Schematic and snapshots of different stimulation protocols. The stimulus perfusion is indicated with a fluorescent solution spiked with Rhodamine B (1% stock diluted with M9 1:7500). In all examples, the animal is loaded with the head toward the top. (a) When the pressure of the stimulus reservoirs (*P_S_*) is equal to the baseline buffer pressure (*P_B_*), or when both the valves are turned off, the animal is not exposed to the stimulus. (b) When the pressure of the stimulus reservoirs is larger than *P_B_*, or when all the valves are open, the animal receives the stimulus at both the head and tail. (c) When the pressure of one stimulus reservoir ( $P_{S1}$) is higher than *P_B_*, or only the upper valve is open, the animal gets stimulated at the head only. (d) When the pressure of one stimulus reservoir ( $P_{S2}$) is higher than *P_B_*, or only the lower valve is open, the animal gets stimulated at the tail only. Scale bar for all images = 100 μm.

Initially, both stimulus reservoirs, linked to the pressure controller, are pressurized at designated values ( $P_{S1},\;P_{S2}$). This initial state must be adjusted to be less or equalize the baseline buffer pressure (*P_B_*), ensuring that no stimulus solution reaches the ends of the trapping channel [Fig. 2(a)]. Once this balance is achieved, control over fluid flow in the stimulator can be attained by activating the valves or, in the absence of valves, by transiently increasing the pressure on one of the reservoirs ( $P_{S1},\;P_{S2}$) during the desired stimulation period. Generally, for stimulation to take place, the pressure of the respective stimulus reservoir must surpass the pressure of the baseline (in our case gravity) flow from the buffer reservoir [Figs. 2(b)–2(d)].

**FIG. 3.**
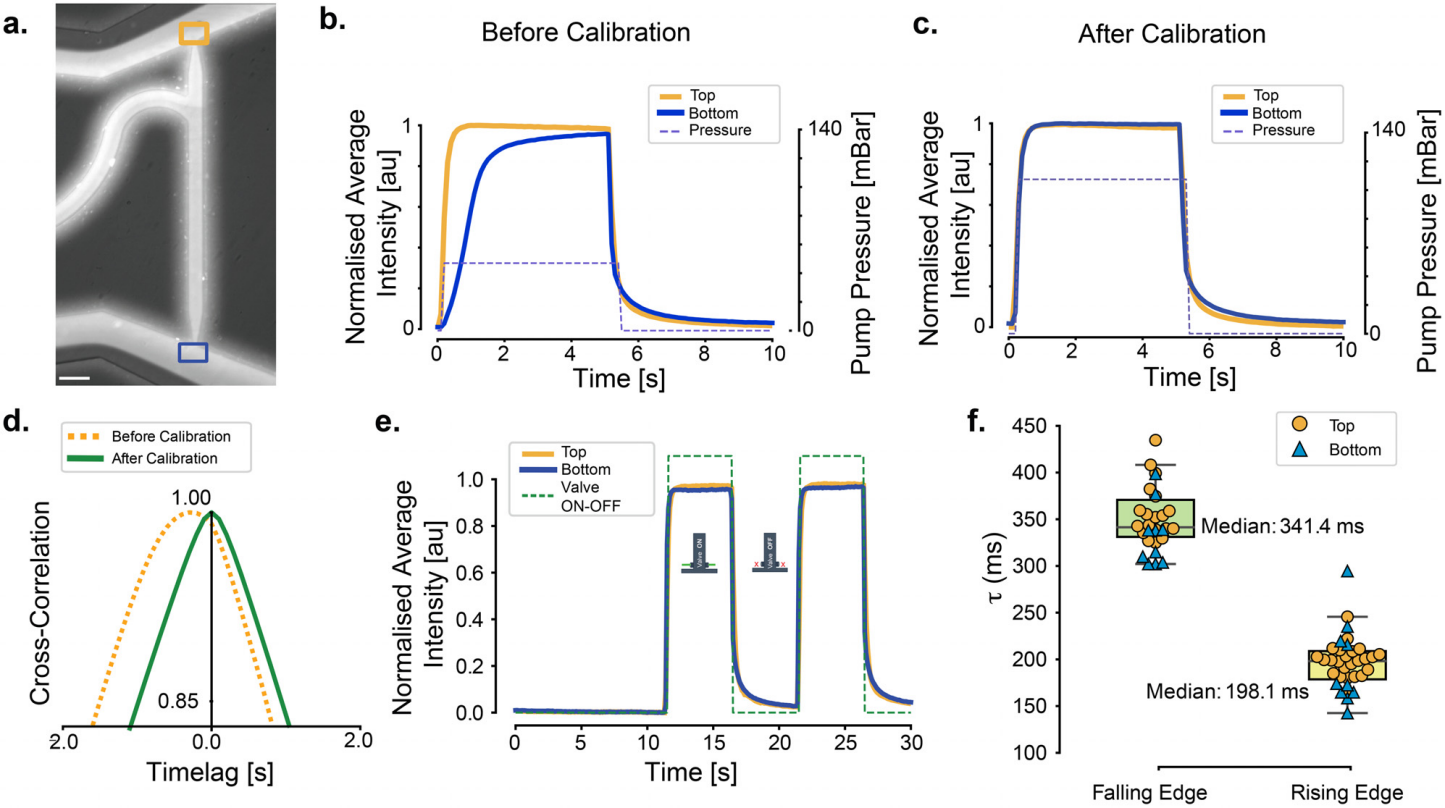
Characterizing the device performance. (a) Simultaneous delivery of a fluorescent dye dissolved in M9 to both head and tail. The mustard and blue squares indicate the measurement of the fluorescent tracer solution at the head and tail, respectively. Scale bar = 100 μm. (b) Representative fluorescence measured at the top (mustard) and bottom (blue) of the trapping channel before calibration, indicating asynchronous delivery. (c) Representative fluorescence after calibration, e.g., adjustment of the pressure on the reservoir recovered synchronization between the top and bottom arrival times. (d) Temporal lag of the stimulus sequence between head and tail, determined from the cross correlation of the intensity signal at the head and the tail entrance (squares in a). The yellow dashed line indicates the lag before calibration of the pressure on the reservoirs, and the green solid line indicates the lag after adjusting the pressure on the reservoir feeding the bottom channel. (e) Time behavior of the (normalized) average fluorescence intensity at the top and bottom exits of the tapered trapping channel, upon opening and closure of the valves. (f) Distribution of response times for the stimulus onset (rising edge) and stimulus offset (falling edge) at the two channels. *τ* was obtained after fitting of the average fluorescence intensity measured for the perfusion at the two trapping channel exits as detailed in the Methods sections.

The precise synchronization of the stimulus delivery to both the head and tail is crucial for accurately studying the temporal response and interneuron integration influencing the organism's behavior. We noted that, however, after setting up the device, the stimulation solution did not necessarily arrive at the head and tail channel exits at the same time. To mitigate this temporal misalignment, we implemented a calibration procedure to be performed before introducing the animal into the experimental setup (see supplementary material Method 3.1). In order to observe the fluid flow, we utilized stimulus solutions containing Rhodamine B and recorded short video clips of channel perfusion [Fig. 3(a), supplementary material Video 1]. In the case of a significant lag between the two flows [Fig. 3(b)], we adjusted the pressure on either reservoirs and repeated the calibration until the delay was minimal [Fig. 3(e)]. Alternatively, it is also possible to set a delay in the valve operation to compensate for the flow lag. The procedure for calibration and its limitations are detailed in the supplementary material Method 3.1. In short, we were routinely able to minimize the delay within the resolution or the camera frame rate (<100 ms) such that they appeared simultaneously at the trapping channel exit.

The fine control provided by the pressure regulation system allowed us to adjust and synchronize the delivery of stimuli, enabling simultaneous stimulation of both the head and tail regions of the worm [Fig. 3(d) and supplementary material Fig. 2]. We found that this calibration procedure is robust between different devices, as long as the reservoirs that deliver the buffer solution by gravity flow are at the same height and filled approximately to the same level, and the length of the connecting tubes does not change.

### C. Dynamics of the stimulus delivery

In order to characterize the speed of stimulus delivery, we quantified the dynamics of the fluorescence intensity at the two exits of the trapping channel [Fig. 3(e)] as a measure of the response time. We quantified the rise and decay time constants (*τ*) of the fluorescence intensity (see supplementary material Method 3.2 and supplementary material Fig. 3) and found an average rise time of ≈200±38 ms [Fig. 3(f)], which completely stabilized after 500 ms. The decay time constant, characterizing the response after stimulus offset, was significantly slower ( τ=350±23 ms). This is due to the lower pressure of the baseline buffer reservoir, which we fed by gravity flow. A faster off-response can be achieved by increasing the pressure of the baseline buffer. Given that the GCaMP6-based neuronal activity sensor has a decay time constant of 1 s (for GCaMP6f) in response to a single action potential (Chen *et al.*, 2012), we infer that our actuator operates faster than the anticipated biological response.

Taken together, a well-calibrated setup of the dual olfactory device allows for simultaneous stimulation of the head and the tail, with a response time of 200 ms.

### D. Imaging neuronal activity in response to head/tail stimulations

**FIG. 4.**
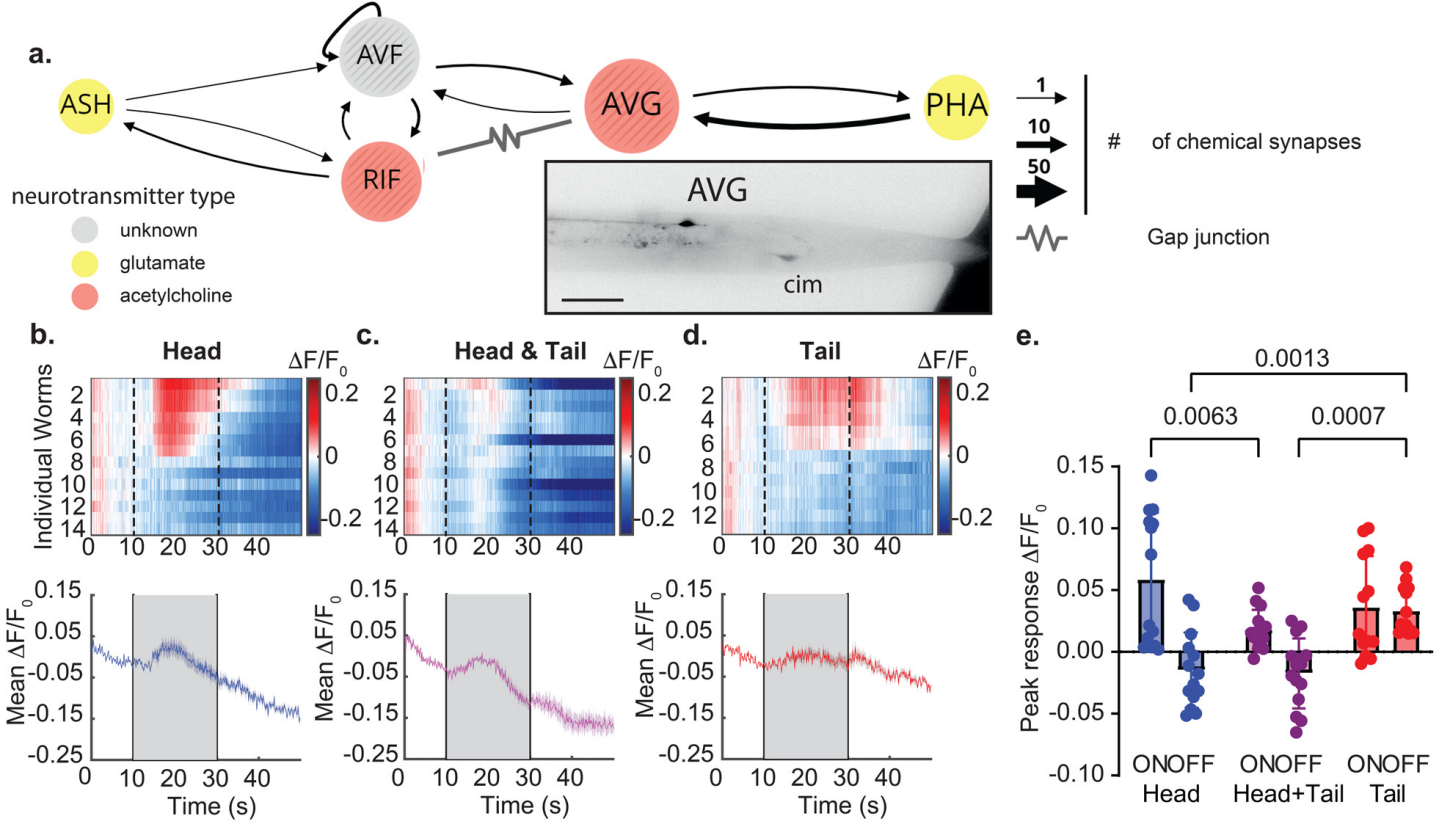
AVG interneuron responses to stimulation at the head and the tail neurons. (a) Schematic representation of the neuronal circuit, showing synaptic connections from the head and tail olfactory neurons into AVG with black arrows. Gap junctions are denoted by zigzag lines. The thickness of the arrows corresponds to the number of synaptic densities identified in the electron microscopy datasets (Witvliet *et al.*, 2021; Cook *et al.*, 2019; and White *et al.*, 1986), which is a proxy for synaptic strength. The color of the nodes indicates the type of neurotransmitter used: yellow, glutamate; red, acetylcholine; gray, unknown. Adapted from nemanode.org (Witvliet *et al.*, 2021). The inset shows a representative micrograph of an AVG:GCaMP6 expressing animal subjected to an olfactory stimulus. Scale bar = 50 *μ*m. cim, coinjection marker. (b)–(d) Calcium transients were recorded from AVG neuronal cell bodies after stimulating the head (b), head and tail (c) and tail (d). The kymographs (upper picture) indicate the normalized and smoothed (see Sec. IV) individual traces from 14, 14, and 13 animals. The heatmaps show the sorted individual animal responses. The lower picture represents the average calcium response, and the shaded area is the standard error of the mean. Dotted vertical lines frame the time of stimulus delivery. (e) Quantification of the peak response at stimulus onset and offset for each of the three stimulation protocols (see Sec. IV). The P-value above the horizontal brackets was derived from a two-way ANOVA analysis, followed by a Tukey's multiple comparisons test.

We chose to investigate how the interneuron AVG processes exposure to hyperosmolarity solutions (2 M glycerol) that elicit an avoidance response in freely behaving animals. AVG is an interneuron that receives indirect input from the head chemosensory neurons ASH, while the tail chemosensor PHA makes direct synaptic connection with AVG [Fig. 4(a)]. These head and tail sensory neurons are sensitive to osmotic pressure (Gat *et al.*, 2023; Zou *et al.*, 2017; Hilliard *et al.*, 2002; and Hilliard *et al.*, 2005) and have been shown to respond robustly to 2 M glycerol. Likewise, we have previously shown that AVG is activated following exposure, as well as removal, of 2 M glycerol, when stimulated at the tail (Salzberg *et al.*, 2020), but data about head stimulation are missing. Whether or not a simultaneous delivery of the aversive stimulus would affect AVG response or if a certain stimulus is dominant over another is not known.

After loading the animals into the device, we imaged the baseline GCaMP6s fluorescence signal of AVG to the buffer solution (unstimulated) before recording three types of predefined sequences of stimulations: head-only, tail-only, or head and tail simultaneous stimulation.

We found that the hyperosmotic stimulation of the nose led to a robust activation of AVG at the stimulus onset (ON response) but not when the stimulus was removed (OFF response). We also observed a noticeable lag between stimulus onset and average AVG signal [Figs. 4(b) and 4(e)]. This lag is representative of the fact that ASH does not form direct connections but is filtered through RIF and AVF interneurons [Fig. 4(a)].

Next, when we stimulated the animals at the tail, we observed a lower average ON response, but a noticeable and significantly higher OFF response compared to the stimulation delivered to the nose [Figs. 4(c) and 4(e)]. In contrast, simultaneous stimulation at both the nose and the tail induced significantly lower average ON responses compared to the stimulus delivered only at the nose. Furthermore, simultaneous stimulation did not elicit the OFF response upon stimulus removal [Figs. 4(b) and 4(e)]. However, microfluidically constrained animals that were mock-stimulated with a buffer that did not contain any osmotically active substance did not display any significant change in AVG activity (supplementary material Fig. S4). This excludes any artifacts, such as drift and network level regulation due to microfluidic immobilization (Gonzales *et al.*, 2019), affecting AVG.

Together our findings imply that while head signals mainly control neural on responses in AVG, tail inputs primarily regulate off responses. More importantly, these findings clearly demonstrate how spatially opposing cues, delivered by our novel device, induce distinct neural responses in the AVG interneuron [Fig. 4(e)]. Another notable feature is the observed variability in the AVG calcium activity, which may reflect the internal state of the neuronal network. This is not a *C. elegans* specific feature but may underlie much of sensory processing and has been observed to be important for the successful execution of behavioral programs (Waschke *et al.*, 2021).

The observation that the overall calcium response in AVG is lower after simultaneous exposure may hint toward a network level regulation that reduces ASH-mediated inputs into AVG. Consistent with this interpretation is the remarkable decrease in basal calcium activity as seen in Fig. 4(c) during simultaneous stimulations.

## III. CONCLUSION

The extent to which animals like *C. elegans*, insects, and vertebrates utilize the collective activity of their chemosensory neurons to interpret olfactory inputs is not well understood (Lin *et al.*, 2023). With advances in microfluidics and imaging, it is now possible to combine high-throughput odorant stimulation with brain-wide imaging and tracking in behaving animals. However, current olfactory chips are limited to sequential stimulus delivery, exposing either the head or the tail of the animal to the odor. Hence, these experimental designs lack the naturalistic features of the environment, where head and tail sensors are exposed to the same stimulus at a time. By stimulating both the head and the tail of the worm, we can more closely replicate the type of chemical exposure the animal would experience in the wild, which allows for a more comprehensive understanding of olfactory perception and behavior. This is achieved through an optimized loading channel geometry, which improves experimental throughput, and precise delivery of stimuli to both the head and tail using microfluidics.

In the future, to obtain real-time monitoring of neuronal dynamics in response to complex stimulus patterns, the device can be integrated with rotary valves to expose the trapped animal to a variety of naturalistic chemical stimuli. In summary, the prospective value of this approach lies in the capability of a microfluidic platform to immobilize a worm, control micro-environments, and observe and record its neural activity automatically. This approach can be further developed to study the functional correlations of activities of sensory neurons, interneurons, and motorneurons in conjunction.

## IV. METHODS

### A. Animal strains and maintenance

Animals were kept and maintained as described by Porta-de-la Riva *et al.* (2012). To record calcium transient in AVG upon stimulation, we used transgenic animals {MOS488, [*him-5;* *etyEx31;lite-1(ce314)*]} expressing GCaMP6s in AVG (Setty *et al.*, 2022).

### B. Mold fabrication

All device designs [Fig. 1(a) and supplementary material Fig. S1] were made in AutoCAD 2022 and converted to CleWin to create the digital mask to fabricate a mold in SU-8 photoresist using photolithography (Xia and Whitesides, 1998) with a maskless aligner (Heidelberg MLA150). First, we cleaned the silicon wafer with propanol and acetone, rinsed it with MQ-water, dehydrated it on a hot plate at 120 °C for 10 min, and let it cooldown at room temperature. Subsequently, we spin-coated 3 ml of SU-8 50 photoresist (MicroChem) on a 10 cm wafer (MicroChem). To obtain a photoresist layer with a thickness of 50 μm, we set the spinning rate at 2000 RPM. Following the coating, we baked the SU-8-coated wafer at 65 °C for 6 min, gradually increasing the temperature (e.g., 8 °C/min) to 95 °C, and allowing it to sit for 20 min. Afterward, the resist was patterned with the design using the MLA150 and then baked for 1 min at 65 °C and 5 min at 95 °C. After allowing the wafer to cooldown to room temperature, we developed it for 6 min (SU-8 developer, Kayaku Advanced Materials) and rinsed it with propanol. Finally, we dried the mold with nitrogen and hard baked it at 120 °C for 2 h.

### C. PDMS preparation

PDMS (polydimethylsiloxane, Dow Corning) was prepared by mixing Sylgard-184 and reagent in a ratio 15:1. The resulting mixture was degassed with a desiccator for 1 h until fully transparent. Before casting PDMS, the mold's surface was vapor-phase silanized with chlorotrimethylsilane gas [ ≥ 99.0% (GC) SIGMA-ALDRICH] inside a chemical fume hood to prevent excessive adhesion of the PDMS to the wafer, thus facilitating the peeling process. PDMS was then poured onto the mold with a thickness of 1 cm and cured in the oven at 85 °C for 90 min. Following the PDMS curing process, inlets and outlets were created with a 0.75 mm diameter biopsy punch (0.035 × 0.025 × 1.5 304 SS TiN coated, SYNEO EUROPE Ltd.).

### D. Chip assembly

We activated the surfaces of a glass coverslip (#1.5 GoldSeal, 24 × 60 mm) and the PDMS with O_2_-plasma for 30 s at 30 W. To enhance adhesion, the two surfaces were placed in contact and annealed on a hot plate for 10 min at 120 °C. Ultimately, we connected the tubes to the inlets and outlets of the chip with hollow stainless steel pins. All the inlets and outlets were connected to 25 gauge polyethylene tubing (PE-25, Phymep) using metal catheter connectors (Phymep, France).

### E. Experimental setup

Before entering the young adult animal into the trapping channel, the inlets were connected to the appropriate buffer reservoirs, while the outlets, through which fluid waste emerges, were directed into waste containers [see chip design in Fig. 1(a)]. The “baseline” non-stimulus buffer was supplied through gravity flow: we positioned the reservoir on a linear motorized stage to adjust the optimal height, and thus, the flow rate [Fig. 1(d)]. If the same baseline buffer is required, a single reservoir can be distributed into the two inlets using a three-way T-connector. While it is possible to deliver the baseline buffer through a syringe pump, we opted for a simple gravity flow, as no specialized equipment is necessary to ensure a steady baseline fluid flow.

The dual olfactory chip was operated using two channels of a single OB1 pressure controller (ElveFlow) that controls the flow of the two stimulus solutions into the microfluidic device. The flow rate of both buffer and stimulus, prior to stimulus delivery, was ≈ 0.005 ml/min. To ensure precise control over stimuli delivery, each stimulus inlet on the microfluidic chip was connected to a distinct stimulus solution reservoir through individual microfluidic smart valves (MUX Wire controller, ElveFlow), as schematized in [Fig. 1(c)]. The operation of the valves can be programmed and controlled remotely (Elveflow Smart Interface, ESI software), thereby avoiding the complication of a dual-layer device design. This configuration provided control over the independent delivery of different stimuli to the head and tail. To administer the stimulus to either the head, tail, or both at the same time, the flow rate of the stimulus was raised to around 0.01 ml/min. This change was applied specifically to one or both of the stimulus inlets by elevating the pressure on the respective reservoir(s), if no valves are available.

### F. Calibration of the stimulus delivery

To characterize the temporal dynamics of the fluid flows during the head and tail stimulation experiments, we first spiked the buffer reservoir with a fluorescent dye (1% Rhodamine B diluted with M9 at 1:7500), to allow one for clear visualization. Afterward, we sequentially imaged the buffer solution's perfusion as it flowed through the device (see supplementary material Methods and supplementary material Video 1). All the imaging experiments were performed on a Leica DMi8 inverted microscope equipped with a Lumencor SpectraX light source using 5% of the full power of the cyan LED and a Hamamatsu OrcaFlash 4.3 sCMOS camera. To estimate the lag between the simultaneous head and tail stimulation, we imaged the entire animal using a 10×/0.3 objective lens and a 100 ms exposure time. The illumination was synchronized to the camera exposure as described by Das *et al.* (2021). The data analysis was conducted using a combination of FIJI (Schindelin *et al.*, 2012) for image processing, Python programming language implemented in Jupyter notebooks for custom analyses, and structured data stored in comma-separated values (CSV) files. For more details on the characterization of the flow profile, see supplementary material Methods and supplementary material Figs. S2 and S3.

### G. Animal loading into the device

To prepare the animals for the experiment, we followed a four-step process, similar to the loading method described in Fehlauer *et al.* (2018).

First, a single animal was placed on a standard nematode growth media (NGM) agar plate (Porta-de-la Riva *et al.*, 2012) without food, allowing it to naturally “clean” itself from bacteria. Second, a drop of S Basal medium was added to the worm to lift it off the agar plate. Third, the worm was drawn into a polyethylene tube (0.76 × 1.22 mm) filled with S Basal medium using a 3 ml plastic syringe by gently pulling the plunger. Fourth, the end of the tube was attached to the inlet pin of the microfluidic chip [Fig. 1(a)] and the worm was injected into the punch-hole of the chip by gently pressing on the syringe's plunger. Finally, the worm was guided into the trap channel by manually controlling the pressure of the syringe (supplementary material Video 2). At the end of each experiment, we flushed out the worm from the trap channel by pressurizing it with the syringe and repeated the process for a new worm.

### H. Recording neuronal activity from living *C. elegans*

Animals expressing GCaMP in AVG interneurons were mounted into the device as described above to visualize neuronal activity secondary to the external olfactory stimulus. To visualize the proper delivery of a stimulus to the worm, Rhodamine B was added only to the stimulus reservoirs. If the worm moved or the flow was incorrect, the file was discarded, and a second trial was performed with the same worm. No more than two trials were done with the same animal. Fluorescence imaging of calcium activity in AVG was conducted with a Zeiss LSM 880 confocal microscope using a 40× magnification water immersion objective lens. The frame rate was 6.667 Hz, the total imaging acquisition was 1 min, and the stimulus was applied for 20 s after 20 s initial recording of baseline neuronal activity.

GCaMP6s fluorescence intensity was analyzed using FIJI (Schindelin *et al.*, 2012). All the files were exported as TIFF files, and regions of interest (ROIs) corresponding to the neuronal somas were manually drawn to best represent the signal. Subsequently, the mean gray values of these ROIs were exported for further analysis. For each worm, the baseline fluorescent levels for the on response (F0_*ON*_) and the baseline fluorescent level for the off response (F0_*OFF*_) were calculated by averaging the mean gray values of 66 frames (10 s) before stimulus delivery or removal, respectively. Then, for each frame, the ΔF was calculated by subtracting F0_*ON*_ from the value of that time point, and the result was divided by F0_*ON*_, to normalize the differences in the fluorescence baseline levels between individuals (ΔF/F0_*ON*_). The first 66 frames in each recording, i.e., those prior to the frames used for normalization, were discarded from the data. Therefore, in the finalized dataset for the on response, there were 50 s of recording, with the stimulus appearing between 10 and 30 s. For the off response, the dataset was normalized using F0_*OFF*_ in a similar manner (ΔF/F0_*OFF*_).

The moving average of each animal's recording data was computed across 7 frames ( ≈ 1 s). Peak response values were calculated as the maximal values during 20 s of stimulation (ON response) or during 20 s after stimulus removal (OFF response). For the on/off peak responses, the ΔF/F0_*ON*_ and ΔF/F0_*OFF*_ datasets were used, respectively.

## SUPPLEMENTARY MATERIAL

See the supplementary material for details regarding four supplementary figures, two supplementary videos, and supplementary description of the methods.

## Data Availability

The data that support the findings of this study are openly available in Zenodo at https://doi.org/10.5281/zenodo.10843377, reference Verburg *et al.* (2023).
